# Painful testicular swelling: an unusual localization of sarcoidosis

**DOI:** 10.11604/pamj.2020.36.222.24749

**Published:** 2020-07-28

**Authors:** Karima Bouytse, Hanane Haddaoui, Jouda Benamour, Jamal-Eddine Bourkadi

**Affiliations:** 1 Pneumo-Phtisiology Department, Faculty of Medicine and Pharmacy of Rabat, Moulay Youssef Hospital, Rabat, Morocco

**Keywords:** Sarcoidosis, testis, scrotal mass

## Abstract

Sarcoidosis is a systemic inflammatory disease characterized by non-caseating epitheloid granulomas; whereas it usually involves the lungs and lymph nodes. Testicular sarcoidosis is extremely rare, having been reported to occur in 0.2% of all sarcoidosis patients. We describe a very unusual form of sarcoidosis of the testis, mimicking malignancy at initial presentation.

## Introduction

Sarcoidosis also known as Schaumann-Besnier disease is an idiopathic granulomatous disorder characterized histologically by non-caseating epitheloid granulomatous lesions that classically affect the chest and lymph nodes. Involvement of the male genitourinary organs, most commonly in the epididymis followed by the testes, is very rare and reported to be < 0.2% in clinically diagnosed cases [1]. The aim of this study was to report an unusual presentation of sarcoidosis and to provide updated information regarding the diagnostic.

## Patient and observation

**Figure 1:**
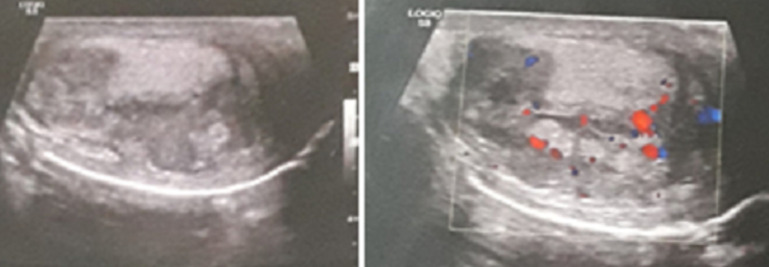
scrotal ultrasonography shows hypoechoic mass lesion in the right testis

A 61-year-old man with no preceding history of genitourinary infections, surgery or trauma and no risk factors for testicular cancer, he denied any contact with tuberculosis patients. He was referred with a 6-month history of right-sided testicular pain and scrotal enlargement witch was progressive, without extra genital signs, notably no respiratory, skin or eye symptoms and no fever. Clinical examination revealed painful and non-tender right testicular mass on palpation with no signs of inflammation at this level. Scrotal ultrasonography showed that the volume of the right testicle is 23ml (25 * 40 * 44mm), with irregular contours, with the presence of hypoechogenic and heterogeneous foci. Color-Doppler showed only subtle intralesional vascularisation (Figure 1). Laboratory examination revealed a white blood cell count of 5,780/mm^3^, lymphocytes of 620/mm^3^, a hemoglobin level of 13.3g/dL; an erythrocyte sedimentation rate (ESR) of 41 mm/h; a C-reactive protein (CRP) level of 9mg/dl (normal, <0.8mg/dl); serum electrolyte levels and renal, liver function test results, were in the normal reference ranges. The 24-h urinary calcium excretion was 5.2mmol/24h (normal, 0-7.5mmol/24h). Tumor markers (serum α-fetoprotein [AFP] and β-human chorionic gonadotropin [β-HCG]) were negative, whereas lactate dehydrogenase (LDH) was at 220IU/L (normal, 135-225IU/L). The tuberculin skin test (TST) was also negative. For three consecutive days, mycobacteria in the sputum and urine were negative.

**Figure 2:**
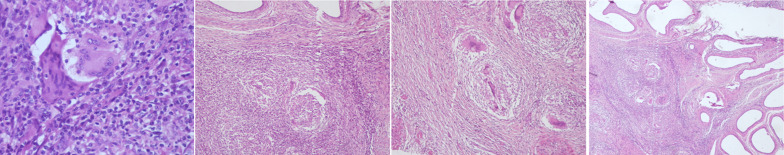
non caseating epitheliod granulomatous lesions of testes

**Figure 3:**
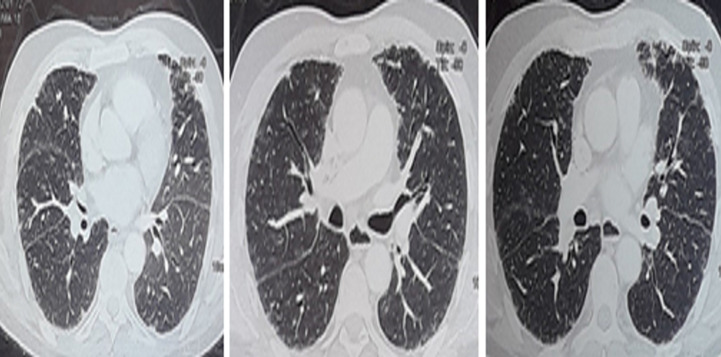
chest CT showed traction bronchiectasis with peripheral reticulations in the upper lobes

**Figure 4:**
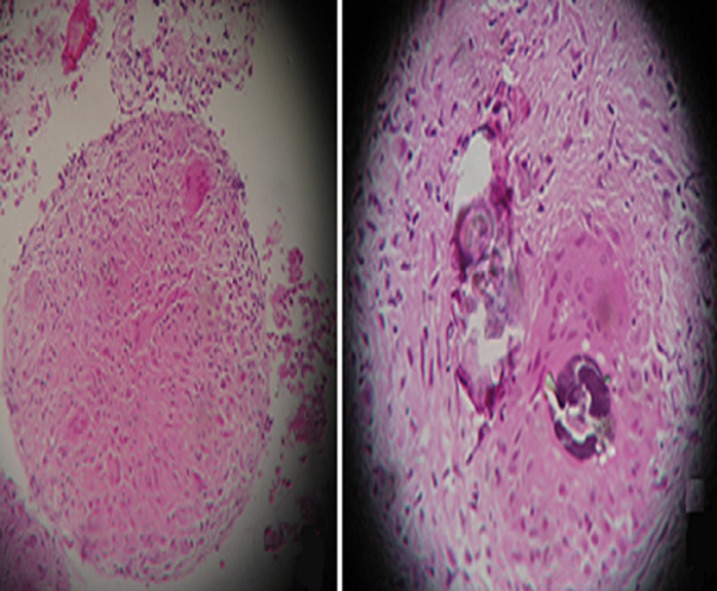
histopathology of the bronchial biopsy: (A) noncaseating epitheloid granulomas; (B) with giant cells containing the shaumann bodies

As malignancy could not still be ruled out, the patient underwent a radical right orchidectomy. Histological examination of the orchidectomy patch showed that the pulp of the testes as well as the siminiferous tubes are partially destroyed and replaced by granulomatous folicular foci of which some areas consist mainly of lymphoplasmocytes, polynuclear neutrophils and eosinophils accompanied by many giant multinucleated cells without casous necrosis, this lesion does not invade the epididymis (Figure 2). Chest CT showed traction bronchiectasis with peripheral reticulations, especially in the upper lobes without visible mediastinal lymphadenopathy (Figure 3). A bronchoscopy was performed to eliminate multifocal tuberculosis (testicular and pulmonary) since tuberculosis is a very common disease in the Moroccan epidemiological context. The histological examination of the bronchial biopsy found non-caseating epitheloid granulomas with the presence of the giant cells containing the shaumann bodies (Figure 4). Bronchoalveolar lavage showed lymphocytic alveolitis (lymphocytes at 47%), GeneXpert MTB/RIF in bronchial aspiration with culture of bronchial fragment in search of tuberculosis mycobacteria were negative. Minor salivary gland biopsy revealed no abnormalities, ophthalmic examination is normal. Further pulmonary work up was consistent with the diagnosis of sarcoidosis: pulmonary function tests showed a slightly decreased total lung capacity suggesting a restrictive lung pattern with normal diffusion capacity. No other vital organs were involved and the patient is in follow up every 4 months. The evolution was favorable after 4 months, the patient was asymptomatic on the respiratory and urinary planes, with a testicular echograhy did not show lesions on the left testicle.

## Discussion

Sarcoidosis is a disease of unknown etiology, characterized by the presence of noncaseating granulomas in multiple organs, the most commonly involved organs are the lungs, the skin and the eyes. The central nervous, muscular, genitourinary and lymphatic systems as well as the heart, salivary glands and joints have been reported to be affected [2]. Genitourinary sarcoidosis, including the epididymis and testis, accounts for less than 0.2% of all clinically diagnosed cases [1]. The most common age and race of patients are 20-40 years old and African-American, respectively, with Asian or Caucasian patients and those older than 40 years being less frequent [3]. Patients with testicular sarcoidosis usually present a nodular, diffuse, painless mass in the unilateral testis [4]. Other symptoms, such as mild pain and scrotal swelling, have been reported [5] as described in our case. Cases of azoospermia due to testicular sarcoidosis have also been described [6]. The major differential diagnosis of a scrotal mass is testicular malignancy. It is important to differentiate sarcoidosis (which is usually a benign and self-limiting disease) from testicular malignancies. The incidence of both diseases peaks in men aged 25-35 years. The initial investigations of a patient presenting with a testicular mass would include tumor markers (AFP, βHCG and LDH). Sarcoidosis is not generally thought to cause false-positive results for AFP or beta-HCG serum levels.

However, there are cases of testicular sarcoidosis with elevated tumor markers in literature [7]; sarcoidosis can cause a raised LDH, which is not a reliable diagnostic test for testicular malignancy. However, AFP and beta-HCG serum levels should be part of the initial assessment of the patient presenting with a testicular mass [8]. For evaluating the scrotal content, ultrasonography is the key imaging modality. Multiple hypoechoic and hypovascular lesions that synchronously affect the epididymis and testis are typical for testicular sarcoidosis. These lesions are rather small (ranging from a few millimeters to 3cm), nodular and sharply demarcated. When bilateral hypoechoic testicular solid nodules are found, the differential diagnosis is wide. Although testicular tumors represent only 1% of all malignant neoplasms in men, it is the most common malignancy in men aged 15-34 years and this is the same age group in which sarcoidosis of the testis is most frequent [9]. In this case, the patient had multiple hypoechoic lesions throughout the right testes. Histological examination remains the most reliable way to confirm the diagnosis of testicular sarcoidosis.

Either by biopsies taken during a conservative approach, recommended when patients have bilateral scrotal masses or there is a strong clinical suspicion of sarcoidosis and the serum testicular tumor markers are negative, or by orchiectomy which is usually performed for the patients presenting with a unilateral testicular mass or those with positive tumor markers [10] as our patient. Many patients with sarcoidosis show spontaneous resolution over a period of up to three years. However, patients with unresolving sarcoidosis are placed on corticosteroid therapy, especially if vital organ function is affected and hypercalcemia is present. In general, corticosteroids have been reported to be effective in reducing the size of testicular lesions and improving symptomatology of sarcoidosis. The amount and period of corticosteroid therapy are controversial and there are few studies on this issue. In cases where corticosteroid therapy fails to control the symptoms or halt the progression of the disease, surgical intervention may become warranted. In addition, methotrexate has been used to treat sarcoidosis. Overall, the treatment of testicular sarcoidosis must be tailored to each patient and the severity of the disease as well as to the preservation of the patient´s fertility if future conception is desired [2].

## Conclusion

Testicular sarcoidosis is very rare, sometimes asymptomatic and characterized by a difficult diagnosis based on clinical, biological and radiological arguments (scrotal ultrasound, chest X-rays and CT chest and abdomen) but the histological confirmation is essential for a final diagnosis.
